# Selective decompression of the ulnar nerve at the arcade of Struthers: A case report

**DOI:** 10.1016/j.jpra.2025.08.027

**Published:** 2025-08-29

**Authors:** Godard C.W. de Ruiter, Michel Wesstein, Karin Boer, Monique H.M. Vlak

**Affiliations:** a Department of Neurosurgery, Haaglanden Medical Center, Hague, The Netherlands; b Department of Neurology, Haaglanden Medical Center, Hague, The Netherlands; c Rehabilitation Medicine, Hand and Wrist Center, Hague, The Netherlands

**Keywords:** Ulnar neuropathy, Cubital tunnel syndrome, Entrapment, Neuropathy, Release

## Abstract

The arcade of Struthers is a known compression site in patients with ulnar neuropathy, but its presence has been debated and in case reports it has mainly been described as incidental finding during cubital tunnel surgery. In this article, we present a case of ulnar neuropathy caused by the arcade of Struthers *only*.

A 27-year-old woman was referred with symptoms of ulnar neuropathy and weakness of intrinsic muscles, but with normal electrodiagnostic tests. On ultrasound (US) an arcade of Struthers was detected proximal to the medial epicondyl with focal indentation of the ulnar nerve and swelling of the nerve proximally and distally. A decompression of the ulnar nerve focused at this site was performed through a small incision in the mid-arm. One year after decompression her pain was almost completely relieved and strength had returned to normal.

This case demonstrates the value of pre-operative US in ulnar neuropathy and the option of selective decompression of the arcade of Struthers.

## Introduction

The arcade of Struthers is a known potential compression site in patients with ulnar neuropathy.^1^ Both its presence and need for exploration during cubital tunnel surgery however have been debated.^2^, ^3^, ^4^ Up to recently the presence of the arcade in patients with ulnar neuropathy has only been reported in a number cases.^2^^,^^5^, ^6^, ^7^, ^8^, ^9^, ^10^ In 2024 a retrospective surgical series from Dr Mackinnon was published, in which the arcade was reported in 30% of the cases that underwent primary surgery for ulnar neuropathy (in which the arcade was detected after transposition of the ulnar nerve).^11^ The present case shows an example of compression of the ulnar nerve by the arcade of Struthers *only*. It demonstrates the importance of detection of this potential compression site in the diagnosis of ulnar neuropathy and the option of selective decompression.

## Case description

A 27-year-old female was referred to our center, because of persistent symptoms of left ulnar neuropathy. The patient had been involved in a car accident in 2020 and had been treated for a communitive distal radius fracture. Soon after the accident she developed symptoms of painful paresthesias radiating from the inside of her left mid-arm across the ulnar side of the elbow and underarm to digits IV and V. The pain scored “8” at the time of presentation on the numeric rating scale (NRS, 0 = no pain, 10 = worst pain ever) despite maximum dose of pregabalin (2dd 300mg).

On neurologic examination there was a positive Tinel sign on the inside of her left mid-arm. She had weakness of her left interosseus muscles and flexor carpi ulnaris muscles. Both muscles graded 4 on the Muscle Research Council Scale (MRC, 0 = no visible contraction, 5 = normal strength). Her grip strength was tested with an electronic hand-held dynamometer (Jamar), which showed 21.1 kg on the left, compared to 33.2 kg on the right side.

Electrodiagnostic (EDX) tests of the ulnar nerve from the referring hospital showed no abnormalities. Needle myography of the abductor digiti minimi, first dorsal interosseous muscles and flexor carpi ulnaris muscles was also normal. EDX testing of the ulnar nerve was repeated at our hospital, which again showed normal findings.^12^ Conduction velocities to the adductor digiti minimus from the lower arm, cubital segment and upper arm respectively were 58, 53 and 60 m/*sec*. Distal motor latency and compound muscle action potential (CMAP) amplitude were 2.5 m/sec and 8.6 mV. Conduction to the first dorsal interosseous muscle showed motor nerve conduction velocities at the lower arm, cubital segment and upper arm of respectively 59, 53 and 60 m/*sec*. Distal motor latency and CMAP amplitude were 2.8 m/sec and 10.1 mV. Needle electromyography showed no abnormalities in the abductor digiti minimi and first dorsal interosseous muscles in the left hand.

**Figure 1:**
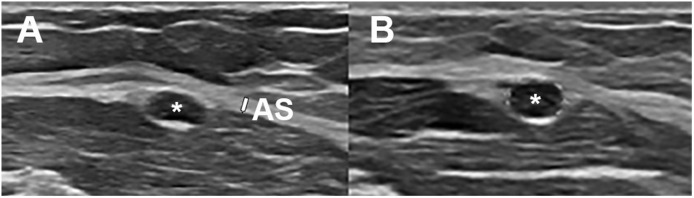
Ultrasound (US) images obtained proximal to the elbow. A: transverse image obtained 5.5 cm proximal to the medial epicondyle. The ulnar nerve (asterisk) passed under a tendinous arcade of Struthers (AS, white arrow) and was oval shaped. B: transverse images obtained just proximal to the arcade of Struthers showed an increase in surface area of the ulnar nerve (asterisk) compared with the level of the arcade.

Because of findings on physical examination an ultrasound (US) was performed with special focus on the left mid-arm. A ligamentous arcade of Struthers was detected 5.5 cm proximal to the medial epicondyle with focal indentation of the ulnar nerve with swelling of the nerve proximally and distally to the arcade, defined as an hourglass-sign. The cross-sectional surface area (CSA) of the ulnar nerve was 7.1 mm^2^ proximal to and 5.5 mm^2^ at the level of arcade (Figure 1). Based on these results of US a selective decompression of the ulnar nerve in the mid-arm could be performed.

**Figure 2:**
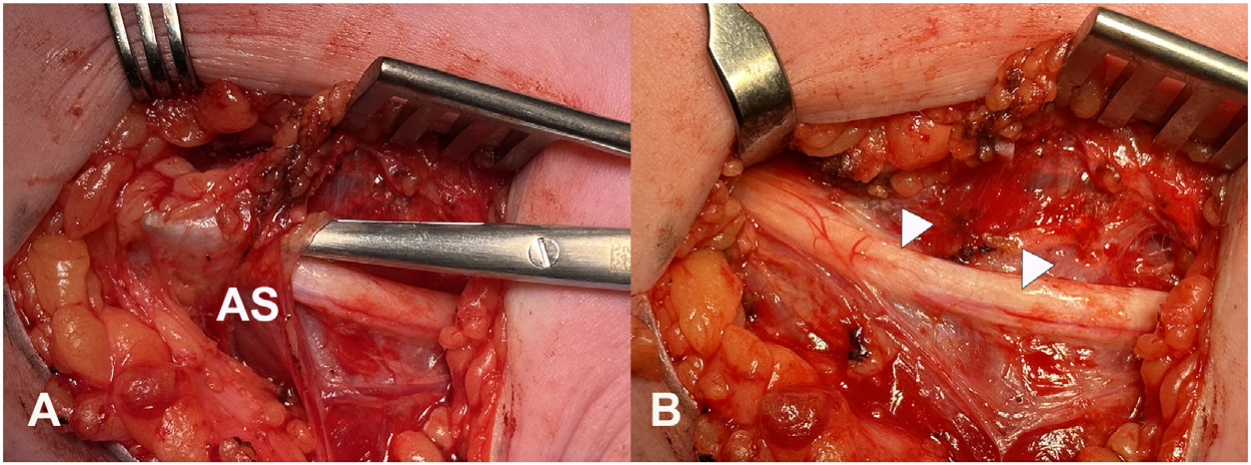
Intraoperative photographs: A: shows the tendinous arcade connecting the medial part of the triceps and intermuscular septum. Jameson scissors have been used to lift up the arcade from the ulnar nerve. Clearly visible is the difference between normal brachial fascia and the thicker arcade (the point of the scissors shine through at the brachial fascia, while the arcade is more dense), B shows the indentation of the ulnar nerve after transection of the arcade of Struthers. Also, clearly visible is the difference in vasa nervorum proximal and distal to the previous site of compression, compared to the previous site of compression (in between white arrows) and the swelling of the nerve proximal and distal to this site (the skin was retracted to show the proximal part of the ulnar nerve).

The patient was operated under general anesthesia without the use of a Tourniquet . The site of incision was marked, with the center 5.5 cm from the medial epicondyle, based on the preoperative measurements of the US. A 5-cm incision was made 8 to 3 cm from medial epicondyle. The ulnar nerve was identified distal to the tendinous arcade of Struthers and followed proximally. At the expected position, there was a clear compression of the ulnar nerve caused by the arcade that was transected in a proximal direction to decompress the ulnar nerve (Figure 2). There were no intra-operative complications. Postoperatively the patient’s symptoms improved with only occasional pain (scored “2” on NRS). One year after surgery the pregabalin was decreased to 2dd 75 mg. US was repeated 3 months after decompression which shows a decreased CSA (5.7 mm^2^) proximal to the previous site of the arcade of Struthers (5.4 mm^2^ at the level of the arcade). Strength on dynamometer had increased to 31.5 kg on the left side 12 months after the surgery.

## Discussion

This case report shows the added value of US in the detection of an arcade of Struthers. Our patient had been evaluated at multiple centers at which the proximal compression site had not been detected with EDX tests.

EDX testing of the ulnar nerve can be challenging. Even at the elbow segment, the most common compression site, nerve conduction measurements are often normal in patients with symptoms suggestive of an ulnar neuropathy (estimated sensitivity ranging from 38 to 89 %^13^). Most centers follow the AANEM guidelines on EDX testing for an ulnar neuropathy at the elbow.^14^ Since this guideline does not include standard measurements of the ulnar nerve at the level of the mid-arm, a neuropathy in this segment will often be missed. Experts have recommended that if EDX tests are negative for an ulnar neuropathy at the elbow, US should be performed and include the entire length of the ulnar nerve.^15^ However not all laboratories have systematically incorporated US into their neurodiagnostic workup. Therefore, inevitably many cases will be missed, in which the ulnar neuropathy is not localized at the elbow. As our case demonstrates an arcade should especially be investigated in patient that had a trauma. The mechanism of injury may not necessarily be the direct impact, but can also be caused by stretching of the ulnar nerve at the site of the arcade of Struthers, similar to the mechanism that has been proposed for ulnar nerve injury in the presence of an arcade of Struthers in base players.^16^^,^^17^ Moreover, careful physical examination should be performed, because, as in our case, a positive Tinel sign on the inside of the mid-arm can also point to this potential compression site.

In addition, our case shows the option of selective decompression of the ulnar nerve in the mid-arm in the case of clear compression by an arcade of Struthers. In the literature, only a small number of cases have been reported, in which with the ulnar nerve was selectively decompressed at this site. Ochiai et al. in 1992 and 2000 reported two cases (out of 3 total) in which the ulnar nerve was decompressed proximally to the sulcus, but in these cases the ulnar nerve was still exposed over extended lengths (as can be observed from intra-operative pictures presented in these articles,^5^^,^^6^ possibly because the compression site pre-operatively had been detected with neurophysiologic analysis). Nakajima also reported a case that was detected with EDX test.^10^ In that case an abrupt change in CMAP was observed 7.5–10 proximal to medial epicondyle. An incision was made from 5 to 12.5 cm above the medial epicondyle to decompress the nerve. Similar to the present case the ulnar nerve had a swollen aspect proximal and distal to the indentation. In comparison to these cases, in our case, a more focused decompression could be performed because the exact location had been determined pre-operatively with US (see Supplemental Figure 1), limiting the size of the incision. With endoscopic procedures this size can be further reduced, because the arcade can be divided through a 2 cm incision placed over the distal end of the arcade.

In the literature, three other cases have been described in detail in which the arcade had been detected with US preoperatively, including two cases previously reported by our group.^7^, ^8^, ^9^ In all three cases the ulnar nerve, in addition to decompression at the level of the arcade, was decompressed at the sulcus. Also, in other cases reported in the literature, including the surgical series from Mackinnon decompression at the level of the arcade was performed after more distal procedures, including decompression and transposition of the ulnar nerve.^11^ As mentioned by the authors this requires removal of the tourniquet,^11^ which probably is one of the reasons why the arcade is not always detected. In the present case the arcade would not have been detected intraoperatively if a tourniquet had been used (see Supplemental Figure 2). Moreover, if the arcade is not decompressed, in transposition of the ulnar nerve this may lead to secondary compression at the level of the arcade (even if this site initially was not the primary compression site). Because of the reasons mentioned above, we prefer to perform US in all patients that undergo surgery for ulnar neuropathy. Besides the arcade of Struthers, also other anatomical variations, such as the epitrochleoanconeus muscle, prominent head of the medial triceps and/or snapping of this part of the triceps, can be detected with US.^18^^,^^19^ In addition, variations of the arcade can be determined, including distinction between a tendinous versus a muscular arcade^20^ and other types that have been distinguished, such as passage through the internal brachial ligament and passage through the medial intermuscular septum.^21^

## Declaration of competing interest

The authors have no conflict of interest related to the information in this article.

## Appendix Supplementary materials

**Supplemental Figure 1** A longitudinal ultrasound (US) image shows an hourglass-sign of the ulnar nerve at the level of the arcade of Struthers (in between white arrows). Intraneural edema was seen over a large area of the nerve. With US the exact location of the arcade could be determined. Image, image 1

**Supplemental Figure 2** A: shows the site for planned incision, which was determined based on preoperative US findings, which showed the arcade 5.5 cm proximal to the medial epicondyle (asterisk). B: shows that with the use of a tourniquet this site could not have been inspected. Image, image 2

## References

[1] Kane E., Kaplan E.B., Spinner M. Observations of the course of the ulnar nerve in the arm. Ann Chir. 27(5):487–96. 4712764

[2] Tang J.B. (2021). Ligament of struthers: exceedingly rarely causes ulnar neuropathy and exploration is not suggested in cubital tunnel syndrome. J Hand Surg Eu.

[3] Bartels R.H., Grotenhuis J.A., Kauer J.M. (2003). The arcade of Struthers: an anatomical study. Acta Neurochir (Wien).

[4] Bartels R.H. (2004). Redefining the "arcade of Struthers". J Hand Surg Am.

[5] Ochiai N., Hayashi T., Ninomiya S. (1992). High ulnar nerve palsy caused by the arcade of Struthers. J Hand Surg Br.

[6] Ochiai N., Honmo J., Tsujino A., Nisiura Y. (2000). Electrodiagnosis in entrapment neuropathy by the arcade of Struthers. Clin Orthop Relat Res.

[7] de Ruiter G.C.W., de Jonge J.G.H., Vlak M.H.M., van Loon-Felter A.E (2020). Ulnar neuropathy caused by muscular arcade of struthers. World Neurosurg.

[8] de Ruiter G.C.W., de Jonge J.G.H., Vlak M.H.M., van Loon-Felter A.E. (2021). Letter to the editor regarding "ulnar neuropathy caused by a muscular arcade of struthers. World Neurosurg.

[9] Sivak W.N., Hagerty S.E., Huyhn L., Jordan A.C., Munin M.C., Spiess A.M. (2016). Diagnosis of Ulnar nerve entrapment at the arcade of struthers with electromyography and ultrasound. Plast Reconstr Surg Glob Open.

[10] Nakajima M., Ono N., Kojima T., Kusunose K. (2009). Ulnar entrapment neuropathy along the medial intermuscular septum in the midarm. Muscle Nerve.

[11] Elmaraghi S., Taylor R., Tung I., Patterson M.M., Mackinnon S.E. (2024). Compression of the Ulnar nerve by the arcade of Struthers: look and you shall find. Hand (N Y).

[12] Kumbhare D.R.L, Buschbacher R. (2015). Buschbacher’s Man Nerve Conduct Stud.

[13] American Association of Electrodiagnostic Medicine AAoN, American Academy of Physical Medicine, & Rehabilitation (1999). The electrodiagnostic evaluation of patients with ulnar neuropathy at the elbow: literature review of the usefulness of nerve conduction studies and needle electromyography. Muscle Nerve.

[14] American Association of Electrodiagnostic Medicine AAo, Neurology AAoPM, & rehabilitation (1999). Practice parameter for electrodiagnostic studies in ulnar neuropathy at the elbow: summary statement. Arch Phys Med Rehabil.

[15] Pelosi L., Aranyi Z., Beekman R. (2021). Expert consensus on the combined investigation of ulnar neuropathy at the elbow using electrodiagnostic tests and nerve ultrasound. Clin Neurophysiol.

[16] Noda I., Fukumoto Y., Kitano M., Kudo S. (2024). Characteristics of ulnar neuropathy in baseball players: focusing on the entrapment point of the ulnar nerve and valgus instability. Shoulder Elbow.

[17] Kawabata M., Miyata T., Tatsuki H. (2022). Ultrasonographic prevalence of ulnar nerve displacement at the elbow in young baseball players. PM R.

[18] Gao J.M., Yuan Y., Gong K.T., Ma X.L., Chen X. (2021). Ultrasound-assisted precise In situ decompression for cubital tunnel syndrome. Orthop Surg.

[19] de Ruiter G.C.W., van Duinen S.G. (2017). Complete removal of the epitrochleoanconeus muscles in patients with cubital tunnel syndrome: results from a small prospective case series. World Neurosurg.

[20] Zhong S., Zhong Z., Yu Y. (2016). Ultrasonic observation and clinical application of arcade of struthers in the mid-arm. World Neurosurg.

[21] Tubbs R.S., Deep A., Shoja M.M., Mortazavi M.M., Loukas M., Cohen-Gadol A.A. (2011). The arcade of Struthers: an anatomical study with potential neurosurgical significance. Surg Neurol Int.

