# Supplementary figures and images for: Selective decompression of the ulnar nerve at the arcade of Struthers: A case report
Source: JPRAS Open. 2025 Aug 29;46:250–4. doi: 10.1016/j.jpra.2025.08.027 (PMC12597039; doi:10.1016/j.jpra.2025.08.027)

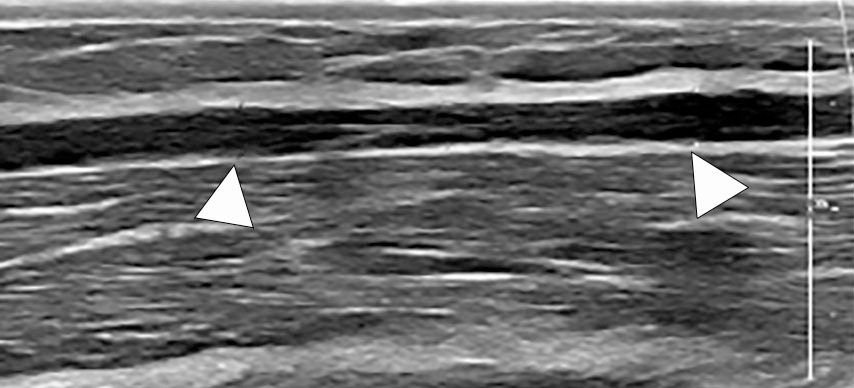

Supplement: Supplementary file 1 — Supplemental Figure 1 A longitudinal ultrasound (US) image shows an hourglass-sign of the ulnar nerve at the level of the arcade of Struthers (in between white arrows). Intraneural edema was seen over a large area of the nerve. With US the exact location of the arcade could be determined. [file mmc1.jpg]

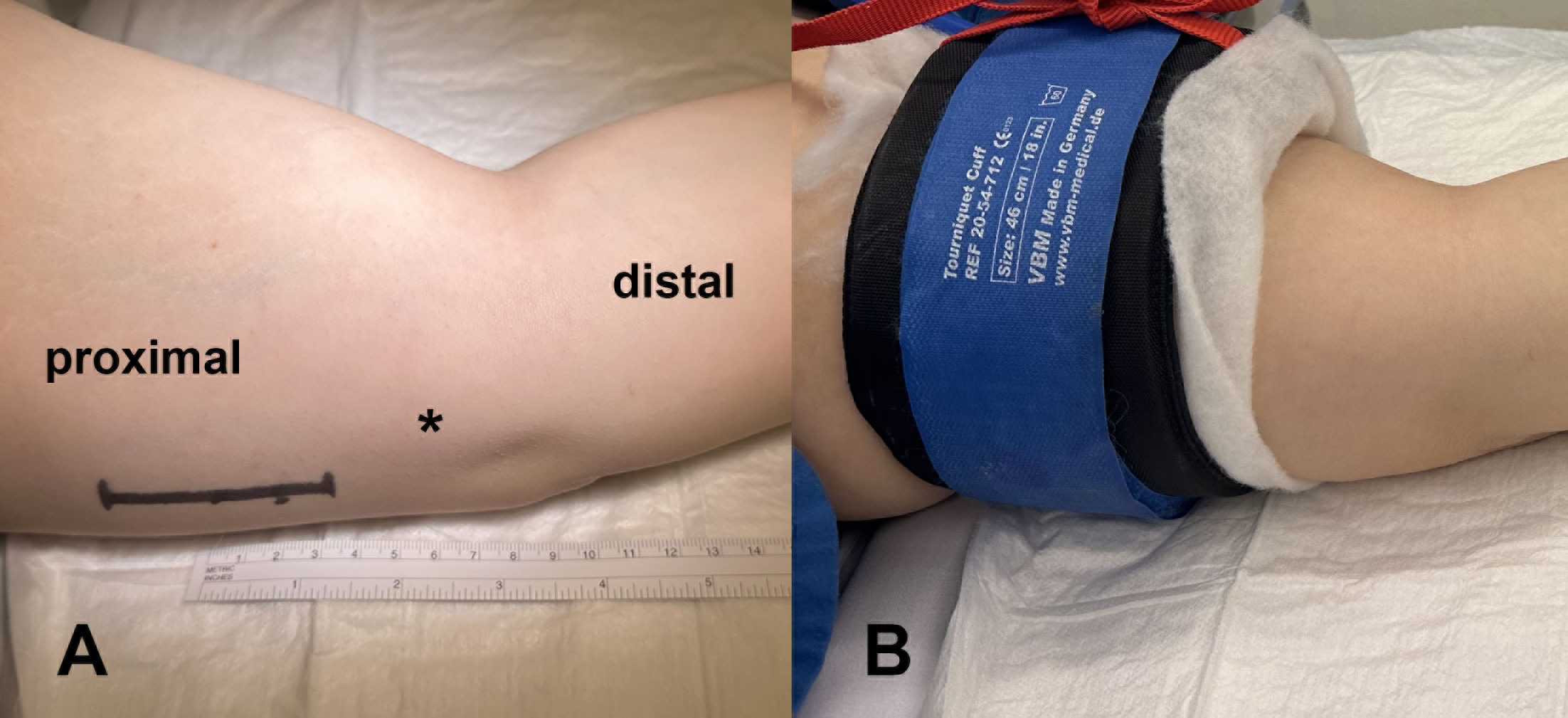

Supplement: Supplementary file 2 — Supplemental Figure 2 A: shows the site for planned incision, which was determined based on preoperative US findings, which showed the arcade 5.5 cm proximal to the medial epicondyle (asterisk). B: shows that with the use of a tourniquet this site could not have been inspected. [file mmc2.jpg]
